# Adoptive immune cell therapy for colorectal cancer

**DOI:** 10.3389/fimmu.2025.1557906

**Published:** 2025-04-01

**Authors:** Chenxiao Liu, Nan Liu, Tongcun Zhang, Yanyang Tu

**Affiliations:** ^1^ Guangdong Province Science and Technology Expert Workstation, Huizhou Central People’s Hospital, Huizhou, Guangdong, China; ^2^ Institute of Biology and Medicine, College of Life and Health Sciences, Wuhan University of Science and Technology, Wuhan, China; ^3^ Science Research Center, Huizhou Central People’s Hospital, Huizhou, Guangdong, China; ^4^ Huizhou Central People’s Hospital Academy of Medical Sciences, Huizhou Central People’s Hospital, Huizhou, Guangdong, China

**Keywords:** adoptive immune cell therapy (ACT), immune cells, colorectal cancer (CRC), tumor antigens, clinical trials

## Abstract

Colorectal cancer (CRC) is a major cause of cancer-related morbidity and mortality worldwide, with limited options for patients at advanced stages. Immunotherapy, particularly immune cell-based therapies, has gained significant attention as an innovative approach for targeting CRC. This review summarizes the progress in various immune cell therapies, including DC vaccine, CAR/TCR-T cells, CAR-NK cells et al, each engineered to recognize and attack cancer cells expressing specific antigens. CAR-T cell therapy, which has been successful in hematologic cancers, faces challenges in CRC due to the solid tumor microenvironment, which limits cell infiltration and persistence. CAR-NK cells, CAR-M and CAR-γδ T cells, however, offer alternative strategies due to their unique properties, such as the ability to target tumor cells without prior sensitization and a lower risk of inducing severe cytokine release syndrome. Recent advances in lentiviral transduction have enabled effective expression of CARs on NK and γδ T cells, providing promising preclinical results in CRC models. This review explores the mechanisms, tumor targets, preclinical studies, and early-phase clinical trials of these therapies, addressing key challenges such as enhancing specificity to tumor antigens and overcoming the immunosuppressive tumor microenvironment. The potential of combination therapies, including immune checkpoint inhibitors and cytokine therapy, is also discussed some as a means to improve the effectiveness of immune cell-based treatments for CRC. Continued research is essential to translate these promising approaches into clinical settings, offering new hope for CRC patients.

## 1 Introduction

According to the latest global cancer burden data for 2020 released by the World Health Organization, there were 19.29 million new cancer cases worldwide in 2020, including 1.93 million cases of colorectal cancer, ranking third. There were 9.96 million cancer deaths worldwide, and colorectal cancer deaths were 940,000, ranking second (1). Colorectal cancer (CRC) is difficult to detect in its early stages, and current treatment options for advanced colorectal cancer include surgery, radiotherapy, chemotherapy (such as fluoropyrimidines, irinotecan and oxiplatin) and targeted therapy (e.g., bevacizumab, EGFR inhibitors and multikinase inhibitors). Even with multiple treatment options, the 5-year survival rate for patients with advanced colorectal cancer is only 20%-30% (2).

**Figure 1:**
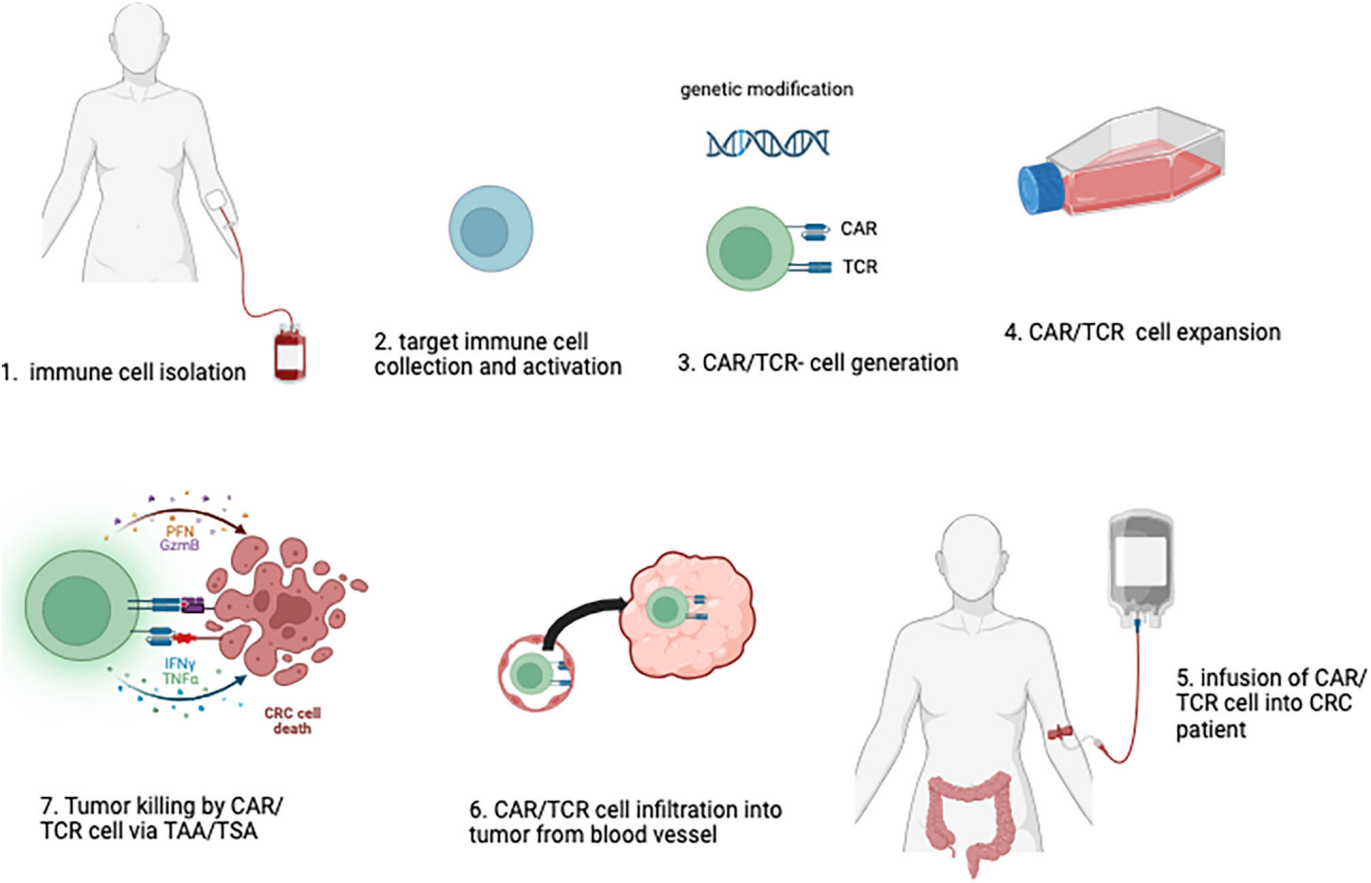
Adoptive Immune Cell Therapy Approach Against Colorectal Cancer. In the adoptive immune cell therapy process, human immune cells are initially harvested (1), selected and activated (2), *in vitro*. If necessary, genetic modifications are introduced to enable the expression of CAR (Chimeric Antigen Receptor) or TCR (T-cell Receptor) on the cells (3). These modified immune cells are then expanded and formulated into a therapeutic product, with rigorous quality control measures in place (4). The final cell product is subsequently administered to the patient (5), where it is designed to target and eliminate tumor cells by tumor homing, and recognizing specific antigens associated with colorectal cancer (6, 7).

Immunotherapy represents a promising class of novel therapeutic strategies in oncology. These therapies include the administration of cytokines to stimulate immune responses, monoclonal antibodies targeting immune checkpoint inhibitors, dendritic cell (DC)-based vaccines, and other immunomodulatory approaches. Among these, adoptive cell therapy (ACT) has emerged as one of the most promising interventions. ACT involves the infusion of ex vivo-expanded and/or genetically modified immune cells, such as T cells or natural killer (NK) cells, which are specifically targeted to tumor sites to enhance anti-tumor immunity. This approach has demonstrated significant potential in the treatment of various malignancies (**Figure 1**).

ACT has evolved significantly since the 1960s. Early research demonstrated that T-cells from cancer patients, especially melanoma, could recognize and kill tumor cells. Steven Rosenberg et al. ‘s pioneering work in the 1980s led to the discovery that lymphokine-activated killer cell (LAK) and tumor-infiltrating lymphocytes (TILs) could be expanded ex vivo and used effectively in therapy (3), and in 1990s, his team significantly advanced the molecular understanding of T-cell responses to cancer and laid the foundation for antigen-targeted immunotherapies (4). These contributions also spanned the development of gene-engineered T-cells, which led to the emergence of chimeric antigen receptors (CARs)-T therapy in 2000s. Later, immune checkpoint inhibitors, such as PD-1/PD-L1 and CTLA-4 inhibitors, enhanced T-cell responses by relieving functional suppression, improving the efficacy of immune cell therapies. By the 2020s, CAR-T cell therapy demonstrated notable success in treating hematologic cancers and began to be extended to solid tumors, with ongoing efforts to overcome immunosuppressive tumor microenvironments and improve treatment strategies (5).

ACT involves the infusion of various immune cell types into patients, including tumor-infiltrating lymphocytes (TILs), peripheral blood-derived immune cells, induced pluripotent stem cell (iPSC)-derived immune cells, and others. The early milestones in the development of ACT were marked by the adoptive transfer of TILs and ex vivo-expanded lymphokine-activated killer (LAK/CIK) cells. Since then, the use of peripheral blood T cells genetically engineered to express transgenic antigen receptors specific to tumor-associated antigens has become a common practice in ACT. Typically, autologous T cells derived from peripheral blood are activated ex vivo and subsequently modified to express transgenic receptors, such as chimeric antigen receptors (CARs) or T cell receptors (TCRs). In recent advancements, gene-modified immune cells undergo a brief *in vitro* expansion phase before being infused into patients, further enhancing their therapeutic potential (6–8). ACT for colorectal cancer has been extensively explored in both basic and clinical research, with several studies demonstrating promising outcomes (9). However, the therapeutic application of ACT remains limited due to numerous challenges encountered in the treatment of colorectal cancer.

In addition to addressing the limitations and the need for further research to make this novel therapeutic approach more widely accessible in clinical settings, our review aims to provide a comprehensive overview of the current state of knowledge regarding adoptive immune cell therapies for CRC, including insights from both preclinical studies and clinical trials.

## 2 CRC classification and gene mutations

Before any therapeutic intervention is considered, cancer staging serves as a foundational tool for assessing the anatomical extent of the disease. The clinical stage, determined based on the patient’s medical history, physical examination, diagnostic biopsies, and imaging conducted prior to the initiation of treatment, reflects the severity of the disease at the time of diagnosis (10). In addition to the clinical stage, a distinct pathological stage—based on post-surgical histological analysis—is assigned to patients undergoing surgery as a treatment option. At the various time points outlined above, both the clinical and pathological stages involve characterizing the extent of the primary tumor (T category), the involvement of regional lymph nodes (N category), and the presence of metastasis to distant sites (M category). Stage groups I, II, III, and IV are determined by combining the T, N, and M categories once they have been assessed. Colon cancer typically grows intraepithelial, initially invading the mucosal lamina propria and submucosa before spreading throughout the entire colon and potentially metastasizing to other organs. The TNM classification system also incorporates tumor deposits in its assessment. Currently, colon cancer is staged into four phases based on the extent of primary tumor invasion (T), the number of regional lymph nodes (N) involved with tumor spread, and the presence of distant metastases (M) (11, 12).

Chromosomal instability, CpG (cytosine nucleotide phosphate guanine nucleotide) island methylator phenotype, and microsatellite instability are major causes of CRC at the genomic level in general. RAS and BRAF mutations in colorectal cancer are often driven by genetic and environmental factors. KRAS mutations typically arise due to chromosomal instability, whereas BRAF mutations, particularly V600E, are commonly associated with MSI and defective DNA repair mechanisms. Both mutations result from accumulated genetic alterations over time, influenced by carcinogens, inflammation, and hereditary predispositions (13). These mutations analyses are usually important, with RAS being a predictive marker for anti-EGFR therapy and BRAF p.V600E mutation serving as a prognostic marker. High-throughput sequencing technologies have introduced novel biomarkers and testing strategies, such as tumor mutation burden (TMB) and liquid biopsy, which can predict immunotherapy response and monitor therapy response when tissue samples are unavailable. However, standardization of these assays and guidelines for their use are needed (14). Commonly, genomic changes related to colorectal tumorigenesis also include a loss of at least one wild-type copy of a tumor-suppressor gene such as APC, P53 or SMAD4 related to chromosomal instability. These genetic losses contribute to the malignant transformation of colorectal cells by disrupting key regulatory pathways involved in cell growth and apoptosis. In fact, the APC gene is often mutated early in CRC progression, leading to the initiation of adenomatous polyps. Similarly, mutations in TP53 and SMAD4 play critical roles in later stages, promoting tumor progression and metastasis sequencing technologies continue to evolve, they are poised to transform clinical practice by offering more precise and personalized treatment options for CRC patients (15, 16).

## 3 Adoptive immune cell therapy strategies

As mentioned before, below, we describe common cell therapeutic approaches directed concretely against CRC (**Table 1**).

**Table 1:** Summary of ACTs.

Types of ACTs	Cell origin	Main cellular or molecular mechanism	Suitable or proposed for diseases	Advantages	Disadvantages
CIK therapy	Patient’s own PBMCs (14)	Cytokine induced tumor-killer cells (NKT, NK and T cells) (14)	\	◆ Simple preparation ◆ Small side effect (15, 16)	◆ Limited curative effect ◆ Large individual differences (15, 16)
CAR-T cell therapy	Patient’s own T cells (usually) (17, 18)	Genetically engineered T cells, Expressing chimeric antigen receptor (CAR), Direct targeting of cancer cells (19)	Hematological malignancies (e.g., B-cell lymphoma, acute lymphoblastic leukemia (20)	◆ Efficiency ◆ Long-lasting treatments (17, 18)	◆ High side effects (e.g., cytokine release syndrome) ◆ High cost due to complex personalized therapy (17, 18)
TCR-T cell therapy	Patient’s own T cells (usually) (21, 22)	Genetically engineered T cells, Expressing specific TCR, Recognize intracellular antigens (21, 22)	Solid tumor malignancies and viral infection-related cancers (21, 22)	◆ Recognize intracellular antigens ◆ Wide range of application (21, 22)	◆ Restricted by HLA and may trigger autoimmune reactions ◆ High cost due to complex personalized therapy (23–26)
NK cell/CAR-NK cell therapy	Patient’s own or healthy donor NK cells (27)	NK cells or expressing CAR NK cells, Kill cancer cells directly through cytotoxicity (27, 28)	Hematological malignancies and solid tumor malignancies (27, 29)	◆ Independent of MHC molecules ◆ Broad-spectrum antitumor activity ◆ Lower risk of GVHD ◆ Lower risk of cytokine release syndrome (CRS) ◆ No individual preparation required (27, 29, 30)	◆ Limited curative effect ◆ Short survival time in the body ◆ of allogeneic NK cells ◆ Limited penetration of solid tumors ◆ The preparation processes are highly technical (27–29)
γδ T cell/CAR-γδ T cell therapy	Patient’s own or healthy donor γδ T cells (31)	γδ T cells or chimeric antigen receptor (CAR) γδ T cells, kill tumor cells through direct cytotoxicity, secretion of cytokines, and regulation of tumor microenvironment (31, 32)	Hematological malignancy and solid tumor malignancies (31)	◆ Independent of MHC molecules ◆ Broad-spectrum antitumor activity ◆ Lower risk of GVHD ◆ Lower risk of (CRS) ◆ No individual preparation required (31–34)	◆ Limited *in vivo* expansion ◆ Inhibition of tumor microenvironment ◆ The preparation processes are highly technical (31–34)
TIL cell therapy	Patient’s own TIL (35)	T cells isolated from tumor tissue. After amplification *in vitro*, it was transfused back to kill tumor cells (35, 36)	solid tumor malignancies (e.g., colorectal cancer, melanoma) (35–37)	◆ Strong specificity ◆ Target tumor mutant antigens ◆ Low risk of antigen escape (37–39)	◆ Preparation is complex ◆ Limited patient availability (40)
Macrophage (CAR-M) therapy	Patient’s own or healthy donor macrophage (41)	CAR-M cells are engineered to enable macrophages to specifically recognize and kill tumor cells, secretion of cytokines, and regulation of tumor microenvironment (42, 43)	solid tumor malignancies (41)	◆ Independent of MHC molecules ◆ Lower risk of GVHD ◆ Lower risk of CRS ◆ Modulation of the tumor microenvironment (41–43)	◆ Short survival time in the body ◆ of allogeneic macrophage ◆ Efficacy to be verified (43, 44)
DC vaccine	Patient’s own DCs (45, 46)	Utilize DC as antigen-presenting cells to activate the immune system and target cancer cells	Hematological malignancy and solid tumor malignancies (45, 46)	◆ Strong specificity ◆ Activate a multi-dimensional immune response ◆ Lower risk of CRS ◆ Long-term immune memory (46, 47)	◆ Preparation is complex and costly ◆ Inhibition of tumor microenvironment (47, 48)

### 3.1 CIK therapy

The cytokine-induced killer (CIK) cells, a frequently studied cell immunotherapy in CRC, are a heterogeneous group of cells obtained from peripheral blood mononuclear cells (PBMCs) stimulated ex vivo with an anti-CD3 antibody and a cocktail of cytokines (17). Briefly, these cells share functional and phenotypic properties with NKT, NK and T cells and are characterized by rapid expansion ex vivo, non-major histocompatibility complex (MHC)-restricted tumor-killing activity, strong antitumour activity and minimal toxicity. It has been observed that the combination of adjuvant chemotherapy with sequential infusions of CIK cells significantly improved the progression-free survival (PFS), disease-free survival (DFS) and OS rates of CRC patients, especially in those with high-risk T4 stage and insufficient duration of chemotherapy (DFS and OS). Moreover, this combination therapy has also been used on mCRC patients, showing good tolerability and a significant increase in OS (18, 19).

### 3.2 CAR-T cell therapy

Chimeric Antigen Receptor T-cell (CAR-T) therapy is an advanced immunotherapy that involves genetically engineering a patient’s own T cells to target and kill cancer cells. The process starts with collecting T cells from the patient, which are then modified in the laboratory to express a synthetic receptor, known as the CAR (20, 49). This receptor is designed to specifically recognize antigens present on the surface of cancer cells. Chimeric Antigen Receptors (CARs) consist of an extracellular single-chain variable fragment (scFv) that serves as an antigen-binding domain, an intracellular signaling domain (typically CD3ζ chain) for T cell activation, and co-stimulatory molecules that enhance T cell responses. T cells genetically engineered to express CARs, which facilitate tumor-associated antigen (TAA) recognition via the scFv and subsequent T cell activation through the intracellular signaling domain, are referred to as CAR-T cells. Once modified, the CAR-T cells are expanded and infused back into the patient’s body. Upon encountering cancer cells, the CAR on the T cells binds to the tumor antigen, activating the T cells to attack and destroy the cancer cells. CAR-T therapy has shown remarkable success in treating certain types of blood cancers, including leukemia and lymphoma, by leveraging the body’s immune system to achieve targeted tumor destruction (21).

CAR-T cell therapies have demonstrated significant promise in the treatment of hematological malignancies over the past decade, with ongoing efforts to expand these therapies to solid tumors (22). However, further research is needed to validate the efficacy of adoptive cell therapy (ACT) in colorectal cancer (CRC) and to optimize treatment strategies, particularly in areas such as tumor-associated antigen (TAA) selection and CAR-T cell dosing (23). Several CAR-T cell therapies targeting antigens such as EGFR (NCT03152435, NCT03542799), NKG2D and NKG2D ligands (NCT03370198, NCT03310008, NCT03692429), CEA (NCT02959151, NCT03682744, NCT02850836), c-Met (NCT03638206), and EpCAM (NCT03013712) have been investigated in patients with metastatic colorectal cancer. These trials currently enroll CRC patients irrespective of their microsatellite instability (MSI) status. Further research is required to assess whether CAR-T cell therapies could benefit patients with either MSI or microsatellite stable (MSS) tumors. These trials aim to provide new therapeutic options for patients with colorectal cancer by leveraging the specificity and potency of -T cell therapies.

In combination therapy, MSS colorectal cancer (CRC) responds poorly to immunotherapy due to an immunosuppressive tumor microenvironment (TME). Combining CAR-T cells with immune checkpoint inhibitors (ICIs) like anti-PD-1 enhances CAR-T persistence and anti-tumor activity (24). Pre-clinical CRC models show improved T-cell infiltration with PD-1 blockade (25). Ongoing trials (e.g., NCT05089266) are evaluating this strategy, though challenges like toxicity remain. Chemotherapy enhances CAR-T efficacy by reducing tumor burden, promoting immune infiltration, and modulating the TME. Agents like oxaliplatin and 5-FU induce immunogenic cell death (ICD), boosting CAR-T recognition. Low-dose chemotherapy also reduces immunosuppressive cells, further improving CAR-T function (26, 27). Radiotherapy enhances CAR-T efficacy by inducing tumor apoptosis, increasing antigen release, and improving TME accessibility. It promotes CAR-T recruitment, upregulates MHC-I and tumor antigens, and enhances targeting (28, 29). CAR-T combined with ICIs, chemotherapy, and radiotherapy offers new strategies for colorectal cancer treatment. However, more preclinical and clinical studies are needed to explore various aspects of mechanism and efficacy.

More details on CAR-T therapy are stated in next chapter (in antigens/targets introduction part).

### 3.3 TCR-T cell therapy

TCR-T cell therapy is an emerging cancer immunotherapy that involves genetically engineering T cells with tumor-specific T cell receptors (TCRs) to recognize naturally occurring antigens and attack malignant cells. Unlike CAR-T therapy, TCR-T targets peptide-MHC complexes, enabling it to address intracellular antigens (tumor cells produce mutant or overexpressed proteins, which are degraded into peptide fragments. These peptides are presented by antigen-presenting cells (APCs) or tumor cells themselves via MHC molecules). And expanding its potential applications, particularly for solid tumors. While promising results have been observed in melanoma and certain other solid cancers, TCR-T faces challenges such as immunogenicity, MHC restriction, and off-target effects (30, 31). Current research focuses on optimizing TCR affinity, developing non-viral transduction methods, and exploring combination therapies with immune checkpoint inhibitors (31). By enhancing specificity and safety, TCR-T holds significant promise for advancing precision medicine and establishing itself as a critical tool in solid tumor treatment. However, the MHC restriction of TCR-T therapy arises from its reliance on T cell receptors (TCRs) recognizing tumor-associated peptides presented by specific MHC molecules. Due to the high polymorphism of HLA genes, a single TCR-T construct is typically limited to patients expressing a compatible MHC allele, such as HLA-A*02:01. This restriction significantly narrows the eligible patient population (32–35). More details on TCR-T therapy are stated in next chapter (in antigens/targets introduction part).

### 3.4 Natural killer cell/CAR-NK cell therapy

NK cell therapy leverages the natural cytotoxicity of NK cells to identify and kill cancer cells based on stress signals or the absence of “self” markers without prior antigen exposure. In detail, NK cells recognize tumor antigens through a diverse family of receptors, enabling immune surveillance and tumor clearance. NK cell receptors are categorized into activating and inhibitory receptors, with their balance determining NK cell activation. Inhibitory receptors, such as KIRs and NKG2A, recognize MHC-I molecules on normal cells, preventing unintended attacks. However, tumor cells often evade immune detection by downregulating the MHC-Is expression. Activating receptors, including NKG2D, NKp30, and NKp46, recognize stress-induced ligands (e.g., MICA, MICB) or tumor-specific proteins upregulated on the tumor cell surface. When activating signals outweigh inhibitory ones, NK cells become activated, releasing perforin and granzymes to kill tumor cells directly. They also secrete cytokines like IFN-γ to amplify the anti-tumor immune response (36–38). CAR-NK cell therapy enhances this process by engineering NK cells with Chimeric Antigen Receptors (CARs) that precisely target specific cancer antigens, improving their ability to locate and destroy tumor cells (39). This combined approach not only boosts the cancer-killing efficiency of NK cells but also lowers the risk of severe side effects, making it a promising option for cancer immunotherapy.

Lingyu Li’s clinical study demonstrates that combining natural killer (NK) cell therapy with chemotherapy significantly improves 5-year progression-free and overall survival rates compared to chemotherapy alone. Several studies (ect.al. NCT03319459, NCT04616196) are ongoing to utilize the combination of natural killer (NK) therapy with cetuximab, a first-line treatment for EGFR-positive mCRC that also interacts with NK cells, triggering antibody-dependent cell-mediated cytotoxicity (ADCC) at clinical level. Another two NKG2D CAR-NK Cell Therapy studies (NCT05213195 and NCT05248048) is recruiting patients with refractory metastatic colorectal cancer. These therapies involve using NKG2D CAR-modified NK cells to target cancer cells and aim to evaluate the safety and efficacy of this treatment​.

### 3.5 γδ T cell/CAR-γδ T cell therapy

Adoptive transfer of γδ T cells has emerged as a promising immunotherapy for CRC. Unlike conventional αβ T cells, γδ T cells possess innate-like properties, enabling them to recognize and target a broad spectrum of tumor-associated antigens without prior sensitization. γδ T cells expressed multiple anti-tumor receptors NKG2D, NKp30 and NKp46 et al. like CD8+ T cells and NK cells. Interesting, the γ9δ2 TCR recognizes over-synthesized phosphoantigens presented by BTN3A1/BTN2A1, while the γδ1 TCR targets lipid antigens presented by CD1c/d in certain cancer cells. These over-synthesized phosphoantigens and lipid antigens result from lipid metabolism disorders, which commonly occur in cancer cells. This recognition might allow γδ T cells to identify and potentially eliminate tumor cells exhibiting such metabolic aberrations. Recent studies have shown that γδ T cells can effectively induce tumor cell cytotoxicity and modulate the tumor microenvironment, enhancing anti-tumor immunity in CRC. Clinical trials (ACT therapies and non-ACT therapies) have explored the safety and efficacy of γδ T cell-based treatments in CRC, demonstrating encouraging results (40–43). By engineering γδ T cells to express CARs targeting specific tumor antigens, this therapy enhances their tumor-homing ability and cytotoxicity, providing a dual mechanism to recognize and destroy cancer cells. One notable study is a phase I/II trial evaluating the safety and efficacy of allogeneic CAR γδ T cells (CAR001) in subjects with relapsed/refractory solid tumors, including CRC. This study involves dose-escalation to determine the maximum tolerated dose and a dose-expansion phase to assess efficacy, including objective response rate, progression-free survival, and overall survival​.

### 3.6 TIL cell therapy

Recently, TIL therapy has shown remarkable success in treating melanoma, with response rates as high as 50-70% (44). Tumor-Infiltrating Lymphocyte (TIL) therapy is a very personalized cancer treatment that uses a patient’s own immune cells to target cancer. TILs are T cells naturally found within a tumor, indicating they can recognize cancer cells. In this therapy, TILs are extracted from the patient’s tumor, expanded in large numbers in the laboratory, and then re-infused into the patient. Once back in the body, these TILs target and attack cancer cells more effectively (45). In a pilot study, Sentinel-node-based adoptive immunotherapy using expanded CD4(+) Th1 lymphocytes showed feasibility, no side effects, and improved survival and tumor regression in advanced CRC patients (46). While some applications in CRC is still under investigation (47, 48) (NCT05902520, NCT06530303, NCT05576077).

microsatellite instability-high (MSI-H) tumors are characterized by a high mutation burden, which increases the likelihood of generating neoantigens that TILs can recognize. As a result, TIL therapy TIL therapy may be suitable for treating colorectal cancer (CRC) patients with MSI-H (50). Despite its potential, TIL therapy for CRC faces several special challenges, optimizing culture conditions and selecting the most reactive TIL subsets are critical areas of research. Identifying patients who are most likely to benefit from TIL therapy is crucial. Biomarkers such as MSI status and PD-L1 expression are being studied to refine patient selection criteria. While there are significant challenges to overcome, ongoing research and clinical trials are continually improving the efficacy and feasibility of TIL therapy.

### 3.7 Macrophage (CAR-M) therapy

In recent years, CAR-M (CAR-macrophages) therapy has emerged as a promising immunotherapy for solid tumors (51). CAR-M cells are engineered to enable macrophages to specifically recognize and kill tumor cells. Studies have shown that CAR-M cells can enhance anti-tumor immune responses by targeting immunosuppressive factors within the tumor microenvironment. Compared to traditional CAR-T therapies, CAR-M cells can provide broader anti-tumor effects by eliminating tumor-associated macrophages and overcoming immune tolerance mechanisms. Current research focuses on improving the persistence of CAR-M cells, enhancing their cytotoxicity, and overcoming the immunosuppressive environment of solid tumors (51–53).

The self-developed humanized anti-HER2 chimeric antigen receptor macrophage (human anti-HER2 CAR-M) has been developed to use an adenoviral vector system to express CAR molecules, which specifically bind to human HER2 antigens to recognize and kill tumor cells (53, 54). Cellular and animal experiments showed that anti-human HER2 CAR M cells have significant anti-tumor efficacy and good safety (53, 54). Anti-HER2 CAR-M cell therapy is currently undergoing phase I clinical trial targeting colorectal cancer and other solid tumors (NCT04660929).

### 3.8 DC vaccine

Dendritic cell (DC) vaccines utilize dendritic cells as antigen-presenting cells to activate the immune system and target cancer cells. This immunotherapy involves isolating the patient’s own DCs, culturing, expanding, and inducing their differentiation, followed by loading them with tumor-specific antigens derived from the patient’s tumor tissue lysate. The activated DCs are then re-infused into the patient to increase the number of functional DCs in the body and stimulate a T-cell-mediated immune response. This enables precise recognition and elimination of cancer cells carrying specific antigens (55, 56). Even in patients with stage III/IV colorectal cancer, this approach has been used in combination with CIK therapy to enhance quality of life (QOL), minimize toxic side effects, and achieve significant improvements in overall survival (OS), disease-free survival (DFS), and progression-free survival (PFS) (57, 58). Additionally, Ishii et al. demonstrated tumor growth suppression in mouse models by combining this immunotherapy with interferon (IFN)-α therapy, using IFN-α gene-transduced tumor cells (59). One Phase II study (NCT00103142) assesses the survival benefits of two cancer DC vaccines in patients with resected colorectal cancer (CRC) metastases. Vaccinated patients demonstrated improved survival compared to an unvaccinated contemporary group (60). Another Phase I trial (NCT02692976) focuses specifically on the feasibility and safety of this approach (60, 61). A third Phase II study (NCT00558051) explores the feasibility and safety of long-term intralymphatic (IL) infusion of autologous dendritic cells (DCs) using implantable delivery ports in nine stage IV colorectal cancer patient’s post-resection. Eight of nine port implantations were successful (62). Together, these trials aim to determine the potential of DC-based immunotherapy to improve treatment outcomes for patients with advanced colorectal cancer (63). This set of clinical trials investigates the safety, feasibility, and efficacy of dendritic cell (DC) vaccination combined with chemotherapy in patients with metastatic CRC (63, 64).

## 4 CRC targets for adoptive immune cell therapy

### 4.1 Antigen classifications

**Figure 2:**
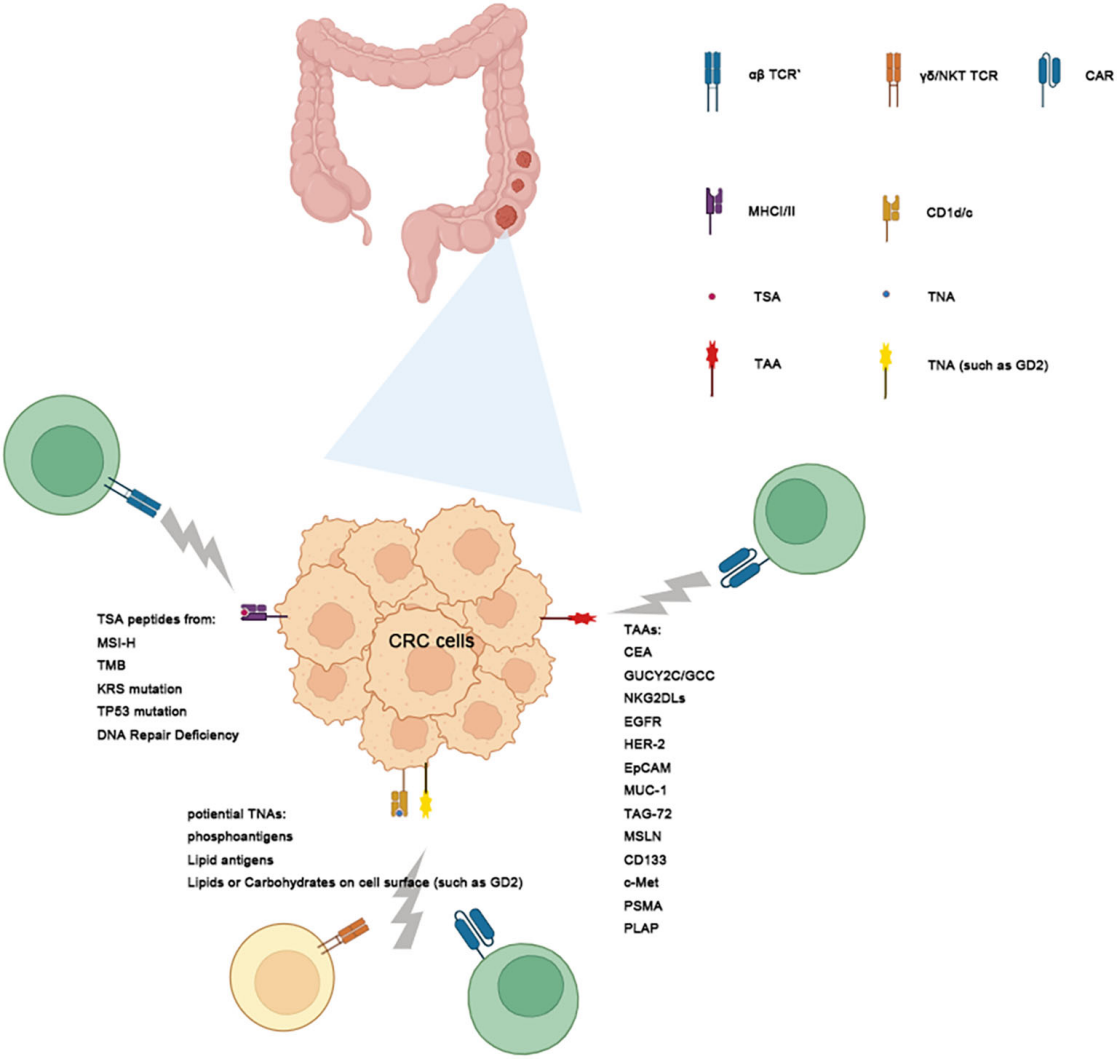
Targeting Tumor Antigens with immunity Cell Therapy. This schematic illustrates the classification of tumor antigens and their role. Tumor antigens are categorized into 3 groups: Tumor-Associated Antigens (TAAs), Tumor-Specific Antigens (TSAs) and Tumor-Non-peptide Antigens (TNAs) in CRC. TAAs: These antigens are expressed in both normal and tumor cells but are overexpressed in tumors. Examples include MUA-1, KKAG-1, and KRAS. While TAAs are common targets for CAR-T therapy, their presence on normal tissues may result in off-target toxicities. Tumor-Specific Antigens (TSAs): TSAs are exclusively expressed in tumor cells and antigen-presented by MCH system, often arising from somatic mutations such as those in KRAS, TP53, and TPAS. These antigens exhibit high specificity, making them ideal targets for TCR-T therapy or DC vaccine with reduced risk of harming healthy tissues. TNAs, including lipids and carbohydrates, are presented by CD1 molecules to activate immune cells like NKT and γδ1 T cells. Gray lightens emanating from the CAR/αβ TCR/γδ TCR/NKTCR indicate its ability to target these antigens, with distinct pathways leading to TAAs, TSAs and TNAs.

A critical challenge in developing Immunity cell therapies for cancer treatment is identifying target antigens that are specific to each cancer type. The choice of targets influences the specificity, efficacy, and toxicity of the therapy, ultimately determining its success. Cancer-derived antigens are classified into tumor-associated antigens (TAAs) and tumor-specific antigens (TSAs), also known as neoantigens, and tumor non-peptide antigens (TNAs) (**Figure 2**).

Tumor-associated antigens (TAAs) are often overexpressed proteins found on tumor cells. While these antigens are relatively easy to identify and are commonly shared among patients through pathological examination, targeting them with CAR-T cells can lead to severe adverse effects due to their limited specificity. In CRC, TAAs include carcinoembryonic antigen (CEA), mucin-1 (MUC-1) et al. Additionally, cancer/testis antigens (CTAs) are highly immunogenic, as they are typically expressed only in testicular cells. Melanoma-associated antigen (MAGE)-A, NY-ESO-1, PASD1, LAGE-1, OIP5, TTK, PLU1, DKKL1, and FBXO39 are among CTAs that are overexpressed in CRC (**Figure 2**, **Table 2**). These antigens are potential targets for immunotherapy due to their selective expression in tumor cells (9).

**Table 2:** TAA summary in CRC.

Targets	Expression/Overexpression	Associated Function	Clinical trials in CAR-T Cell Therapy Research
CEA(CEACAM5)	~50%-98.8%	Unclear	NCT02349724 NCT02959151 NCT03682744 NCT04348643
GUCY2C (GCC)	~95%	Intestinal homeostasis regulation	NCT06675513
NKG2DLs	No specific data was found	External signals under stress or pathogens	NCT03692429 NCT03018405 NCT04107142 NCT04270461 NCT04550663
EGFR	~22.4%	Regulation of proliferation, survival, and differentiation	NCT03152435 NCT03542799
HER-2/HER-3	HER-2: 2–11% HER-3: 75%	Promotion of proliferation, angiogenesis, and metastasis	NCT02713984 NCT03740256
Encamp	90%	Involved in cell adhesion, proliferation, migration, and invasion	NCT03013712 NCT05028933
TAG-72	~80%, (elevated serum levels in ~43% of patients)	Cell adhesion, epithelial integrity, signaling regulation	NA for CRC
MSLN (Mesothelin)	50-~60%	Enhancement of cell proliferation and survival	NCT04503980
MUC-1	~60%	Protection, lubrication, signaling, immunity, proliferation, repair.	NCT02617134
PLAP	~20%	Enzyme	NA
CD133	15.3%	Stemness, regeneration, membrane integrity,	NCT02541370
c-Met	~15%	Regulation of proliferation, motility, and metastasis	NCT03638206
PSMA	75–85%	Enzyme	NA for CRC

Conversely, non-synonymous somatic mutations, including single-nucleotide variations, insertions/deletions, structural alterations, frameshift mutations, and fusion genes, lead to the production of defective peptides (9), referred to as tumor-specific antigens (TSAs) or neoantigens. These neoantigens are potential targets for immune recognition (65). These antigens are often presented by MHC system on surface of tumor cells and recognized by TCR of αβ T cells, but they are difficult to be identified with routine pathological examination. Since TSAs are exclusively expressed in tumor cells, they have high therapeutic value due to the low risk of on-target off-tumor effects (65) (**Figure 2**).

TNA are non-protein molecules synthesized by tumor cells that play a critical role in immune recognition and cancer immunotherapy. Unlike peptide antigens, which are derived from protein fragments, non-peptide antigens primarily encompass lipids, glycolipids, carbohydrates, and certain small molecules unique to or over synthesized in cancer cells. These antigens often arise due to alterations in cellular metabolism and biosynthetic pathways that are characteristic of malignant transformation. The presentation of tumor non-peptide antigens to the immune system typically occurs through specialized antigen-presenting molecules such as the CD1 family, which can present lipid-based antigens to natural killer T (NKT) cells, γδ1 T cells and other immune effectors (66–68). Recent research on tumor non-peptide antigens in colorectal cancer (CRC) highlights promising advancements in immunotherapy approaches (69–73). But most current gene modified T cells therapies focus on CAR-T therapy targeting TAAs due to the low prevalence of these specific mutations and among CRC patients. And some gene modified immune cells therapies focus on TCR-T therapy or DC vaccines therapy targeting TSAs (**Figure 2**).

### 4.2 TAA

Tumor-associated antigen (TAA) is usually highly expressed on tumor cells, which is an important target for adoptive immune cell therapy of colorectal cancer (**Table 2**). The fetal glycosylphosphatidylinositol-anchored glycoprotein CEA, also known as CEACAM5, is a member of the immunoglobulin superfamily. Although the exact function and signaling mechanisms of CEA remain unclear, it is typically undetectable in normal adult tissues, except in the gastrointestinal system, where it is expressed at low levels during the early stages of human embryonic and fetal development (74). It is overexpressed in ~50%- 98.8% of colorectal cancer (CRC) tissues, making it a valuable diagnostic and prognostic tumor marker as well as a promising target for novel CRC treatments (75, 76).

Guanylyl cyclase 2C (GUCY2C or GCC), a member of the mucosal cyclase receptor family, is predominantly expressed in intestinal epithelial cells ranging from the duodenum to the rectum, with the exception of certain hypothalamic neurons (77). It converts GTP to cGMP upon activation by guanylin and uroguanylin. This cGMP signaling regulates fluid secretion, intestinal barrier integrity, and epithelial cell proliferation, differentiation, and apoptosis. GUCY2C also influences systemic energy balance and appetite control. Dysregulation of this signaling pathway can contribute to conditions such as cancer, bowel transit disorders, and inflammatory bowel disease. During the early stages of tumorigenesis, the loss of GUCY2C-binding ligands (guanylin or uroguanylin) leads to the silencing of this pathway, thereby promoting intestinal remodeling (78, 79). GUCY2C is overexpressed in nearly 95% of CRC cases, including metastatic CRC (mCRC), as well as in pancreatic and some gastroesophageal tumors (80).

NKG2D (Natural killer group 2 member D) ligands (NKG2DL) include the cytomegalovirus UL16-binding proteins (ULBP1-6) and MHC-I Chain-related molecules (MIC-A and MIC-B) in humans. These ligands are expressed response to external signals such as stress or pathogens and during neoplastic cell transformation, but are rarely present in healthy tissues (80). NKG2D is a C-type lectin-like transmembrane receptor primarily expressed on NK cells, as well as on CD8+ T cells, γδ T cells, and certain autoreactive or immunosuppressive CD4+ T cells (81, 82). This receptor activates immune cells through the adaptor molecule DAP10/12 and its binding to NKG2DLs, stimulating proliferation, production of pro-inflammatory cytokines, and cytotoxic function. NKG2DLs are expressed in various tumors, including carcinomas (CRC et al), leukemia, lymphoma, multiple myeloma, melanoma, glioma, osteosarcoma, and neuroblastoma (83). Immune cells are capable of recognizing and eliminating CRC cells through the interaction between NKG2D and NKG2D ligands (NKG2DLs) (83). However, the data regarding the overexpression of NKG2DLs on cancer cells have not been found by the authors.

Epidermal Growth Factor (EGFR) is a transmembrane glycoprotein and a member of the protein kinase superfamily. Ligand binding induces receptor dimerization, which subsequently activates signaling pathways that promote cell division, survival, and proliferation (84, 85). Recent studies have identified novel roles for EGFR in regulating autophagy and cellular metabolism, which are activated in response to environmental and cellular stresses in cancer cells (86–88). EGFR is often overexpressed and/or mutated in most solid tumors, including colorectal, brain, renal, ovarian, breast, head-and-neck, and non-small-cell lung cancers (89, 90). Oncogenic mutations, including EGFR variant III (EGFRvIII), which seems to be expressed only in tumor tissue, are the specific target of some immunotherapies. This mutation allows for ligand-independent activation and is found in the extracellular domain of EGFR (91, 92). It has been reported that EGFR expression was detected in 44.7% of cancer tissues compared to 21.4% in normal tissues. Notably, high expression of EGFR was observed in 22.4% of cancer tissues, while no high expression (0%) was found in normal tissues (93).

Human epidermal growth factor receptor 2 (HER-2), also known as ERBB2, a member of the EGFR family, is a transmembrane receptor tyrosine kinase involved in cell growth and differentiation. It is overexpressed in various cancers (including CRC), particularly breast cancer. HER-2 activation through dimerization with other EGFR family members (HER-3) promotes signaling pathways that drive tumor progression, including proliferation, survival, and metastasis (94, 95). HER-2 is overexpressed in CRC with expression rates ranging from 2% to 11%, and both HER-2 and HER-3 are overexpressed in liver metastases of CRC patients (8% and 75%, respectively), making them promising targets for CAR-T therapies (96, 97).

The epithelial cell adhesion molecule (EpCAM) is a type I transmembrane glycoprotein primarily found on the basolateral membrane of normal epithelial cells, where it mediates cell-cell adhesion and regulates epithelial integrity (98, 99). Under normal conditions, EpCAM not only participates in cell-cell adhesion but also regulates differentiation in progenitor and embryonic stem cells (100). However, in cancer, EpCAM overexpression is associated with increased proliferation, migration, invasion, and tumor metastasis (101). In CRC, EpCAM is reported that overexpressed in more than 90% of tumor cells (102).

Mucin-1 (MUC-1) is a heavily glycosylated transmembrane glycoprotein primarily expressed on the surface of epithelial cells. It plays a critical role in protecting mucosal surfaces, mediating cell signaling, and facilitating cell adhesion (103). In cancer, MUC-1 is often overexpressed and aberrantly glycosylated, contributing to tumor progression, metastasis, and immune evasion. Its altered expression in various cancers, including breast, ovarian, and lung cancers, makes MUC-1 a significant biomarker for cancer diagnosis and prognosis (104, 105). MUC-1 expression was observed in 61% of colorectal cancer (CRC) tissues; however, no significant overexpression was detected (106).

The membrane-bound glycoprotein known as tumor-associated glycoprotein (TAG)-72 is normally absent from most normal tissues, with the exception of fetal tissue and the endometrium during the secretory phase (107, 108). It is a mucin-like protein, which contributes to its role in tumor progression, metastasis, and immune evasion and expressed in various epithelial tumors, including colorectal, ovarian, and breast cancers. Compared to normal mucosa, TAG-72 is overexpressed in 80% of colorectal cancers (CRCs), and serum levels are 43% higher in CRC patients (109, 110).

Mesothelin (MSLN) is a glycosylphosphatidylinositol-anchored cell surface protein involved in various aspects of cancer biology, including promoting cell adhesion, proliferation, and survival. However, its precise biological functions are not yet fully understood (111). Normally, MSLN is normally expressed in limited tissues, primarily in the mesothelial cells lining the pleura, peritoneum, and pericardium. It is also expressed in various normal epithelial tissues, including the pancreas, ovaries, and some cells of the lung (112). In cancer, MSLN is overexpressed not only CRC but also in mesothelioma, ovarian cancer, pancreatic cancer, as well as in cervical, endometrial, biliary cancers, uterine serous carcinoma, cholangiocarcinoma, and pediatric acute myeloid leukemia (112, 113). In CRC, up to 50%-60% of malignancies over express MSLN (106, 114).

Cluster of differentiation 133 (CD133), or prominin-1, is a pentaspan transmembrane glycoprotein expressed on membrane protrusions. It serves as a stem cell marker, playing crucial roles in cancer progression, tumorigenesis, and cell differentiation, particularly in hematopoietic and neural stem cells (115). CD133 is expressed by several progenitor and stem cells and plays a role in plasma membrane architecture. In cancers of the kidney, brain, colorectal, lung, pancreatic, and ovarian tissues, it has been proposed as a surface marker for cancer stem cells (116). Higher rates of distant metastasis, metastatic recurrence, and chemoresistance are all linked to CD133 expression in colorectal cancer (117). A study showed 15.3% positive rate in CRC tissues (118).

A receptor tyrosine kinase called mesenchymal–epithelial transition factor (c-Met) is essential for cellular functions like growth, differentiation, and survival. It is expressed in a variety of cell types, including fibroblasts, hematological cells, keratinocytes, endothelium, liver cells, and epithelial cells. It is activated by its ligand, hepatocyte growth factor (HGF) (119) (120). Colorectal, gastric, renal, lung, pancreatic, ovarian, prostate, and breast malignancies all overexpress c-Met. About 15% of individuals with colorectal cancer have overexpressed c-Met, which is linked to cell proliferation, differentiation, cancer progression, and metastasis (119).

Usually found on prostate epithelial cells, prostate-specific membrane antigen (PSMA), also referred to as folate hydrolase I or glutamate carboxypeptidase II, is a transmembrane protein that is also expressed in the duodenal mucosa, salivary glands, some renal tubular cells, and a subset of neuroendocrine cells in colonic crypts (121). In CRC, PSMA is expressed in approximately 75–85% of primary tumors and metastases (122, 123).

Phosphoric acid monoesters are hydrolyzed by the metalloenzyme placental alkaline phosphatase (PLAP). While predominantly expressed in the placenta, PLAP is also detected in trace amounts in other tissues, including the lung, testis, fallopian tubes, and uterine cervix (124, 125). PLAP is overexpressed in CRC and is detected in over 20% of colorectal adenocarcinomas (126).

### 4.3 TSA (neoantigens)

Advances in next-generation sequencing (NGS) have enabled the identification of CRC neoantigens, which are predominantly generated by DNA mismatch repair (MMR) deficiencies and microsatellite instability (MSI)tumors exhibit a high mutation burden, increasing the likelihood of neoantigen formation (127, 128). Neoantigen-based therapies, such as personalized cancer vaccines and adoptive T-cell therapies, have demonstrated potential in preclinical studies and early-phase clinical trials. Moreover, immunity inhibitors (e.g., anti-PD-1/PD-L1 therapies) show enhanced efficacy in MSI-high CRC due to the immunogenic tumor microenvironment fostered by neoantigens (129, 130). However, challenges neoantigen heterogeneity and immune escape mechanisms. Efforts to improve computational algorithms for neoantigen prediction and the integration of combinatory therapies are underway to enhance clinical outcomes.

Linnebacher M et al. have successfully developed cytotoxic T lymphocytes (CTLs) capable of recognizing HLA-A2.1-restricted frameshift peptides through the study of specific frameshift-derived antigens. Among the 16 predicted frameshift peptides, 3 demonstrated the ability to specifically lyse target cells loaded with their corresponding peptides (131). Yu et al. identified various neoantigen-containing peptides, such as SEC11A-R11L and ULK1-S248L, through cytotoxicity assays using neoantigen-reactive T cells (NRTs) in HLA-A0201+PW11 cell models. These peptides demonstrated superior efficacy in eliciting antigen-specific cytotoxic T lymphocyte (CTL) responses compared to their natural peptide counterparts (132). Moreover, Mutations at codon 12 of the Ki-ras gene are frequently observed in pancreatic and colorectal cancers. Analysis of T-cell immune responses revealed that 75% of pancreatic cancer patients and 35% of colorectal cancer patients exhibited specific responses to mutated Ki-ras-derived peptides. Although T cells in individual patients may not recognize the mutated Ki-ras peptides expressed in their own tumors, these peptides show potential as targets for cancer immunotherapy (133).

## 5 Challenges and perspective

The tumor microenvironment (TME) in CRC presents various challenges that can reduce the effectiveness of immune cell therapies, including CAR-T and other APC immune cell treatments. The main immunosuppressive mechanisms, including hypoxia, tumor immune microenvironment (TIME), immunosuppressive cytokines, and the tumor stromal barrier, and we discuss potential strategies to counteract these challenges and improve the therapeutic outcomes for CRC.

Hypoxia is a common feature in solid tumors due to rapid cell proliferation and inadequate blood supply. In CRC, hypoxia is associated with an increase in hypoxia-inducible factors (HIFs), which drive metabolic reprogramming and support an immunosuppressive environment (134). Impact on Immune Cells, Hypoxia reduces the function and persistence of cytotoxic immune cell. For instance, low oxygen levels reduce the effectiveness of glycolysis-dependent T cells, weakening their cytotoxic response (135). Approaches to mitigate hypoxia include using HIF inhibitors, such as acriflavine (136), to block HIF activity, as well as engineering CAR/TCR immune cells to resist hypoxic stress. Genetic modifications enabling CAR/TCR cells to express enzymes that mitigate hypoxia-induced acidity, such as carbonic anhydrase IX, have shown promise (137, 138).

The tumor immune microenvironment (TIME) in CRC can be classified into three distinct subtypes based on cytotoxic T cell landscape: inflamed (“hot”), immune-altered (excluded and immunosuppressed), and immune-desert (“cold”) tumors (139). Inflamed CRC, commonly associated with MSI-H) tumors, is characterized by a robust infiltration of CD8+ T cells, Th1 cells, and dendritic cells (DCs), along with high levels of pro-inflammatory cytokines such as IFN-γ and TNF-α. These tumors typically exhibit strong responses to immune checkpoint inhibitors (ICIs) which widely are expressed on tumor cells as well as tumor immune cells (140–142). Immune- altered CRC displays a substantial presence of T cells; however, these cells are largely confined to the tumor periphery and fail to infiltrate the tumor core. A significant proportion of microsatellite stable (MSS) CRCs fall into this category. Immune-desert CRC, which comprises the majority of MSS CRC cases, is characterized by a paucity of T cells within the tumor due to low tumor mutational burden (TMB), impaired antigen presentation, and an immunosuppressive tumor microenvironment (139). In both immune-excluded and immune-desert tumors, M2 macrophages, recruiting regulatory T cells (Tregs) and myeloid-derived suppressor cells (MDSCs) play key roles in immune suppression. They play a significant role in creating an immunosuppressive microenvironment by secreting cytokines such as TGF-β, IL-10, and VEGF (143). Moreover, M2 macrophages contribute to immune suppression by enhancing tumor angiogenesis and promoting fibrosis, further inhibiting T cell infiltration (142). Tregs and MDSCs inhibit the activation of cytotoxic T cells and reduce the overall anti-tumor immune response.

CRC tumors (tumor cells, Cancer-Associated Fibroblasts (CAFs) and the immune cells) release a range of immunosuppressive cytokines, including TGF-β, IL-10, and VEGF. These cytokines further contribute to an immunosuppressive environment by recruiting regulatory T cells (Tregs) and myeloid-derived suppressor cells (MDSCs), which inhibit the cytotoxic function of effector immune cells (130–132). Immunosuppressive cytokines reduce engineering CAR/TCR immune cells efficacy by decreasing their proliferation, persistence, and cytokine release (133). To improve the effectiveness of CAR-T therapy/other ACT in the tumor immune microenvironment (TIME) of CRC, key strategies can be employed. These include modifying CAR-T cells to resist the suppressive effects of immunosuppressive cells and cytokines. One effective approach is to make CAR-T cells resistant to immunosuppressive cytokines such as TGF-β and IL-10. Alternatively, combining CAR-T therapy with TGF-β inhibitors or other drugs may help enhance the infiltration and activity of CAR-T cells within the tumor, thereby improving treatment efficacy (137, 138). Moreover, combining engineering CAR immune cells therapies with checkpoint inhibitors (e.g., anti-PD-1/PD-L1 antibodies) can help counteract the suppressive cytokine environment (134–136).

The tumor stroma in CRC is a dense structure composed of extracellular matrix (ECM), fibroblasts, and various stromal cells. This dense ECM physically restricts immune cell infiltration and limits the access of CAR-T cells to tumor cells (144, 145). The stromal barrier prevents effective immune cell infiltration and reduces the efficacy of immune cell therapies. Cancer-associated fibroblasts (CAFs) contribute to this barrier by producing ECM components like collagen and fibronectin (146). Stromal remodeling strategies, such as targeting CAFs with fibroblast activation protein (FAP) inhibitors or using hyaluronidase to degrade hyaluronic acid, can enhance immune cell penetration (147–149). Another approach involves modifying CAR cells or other APC immune cells to express enzymes that degrade ECM proteins, which has shown promise in increasing APC cell infiltration in solid tumors (149, 150).

In summary, the immunosuppressive tumor microenvironment in CRC is a major obstacle to effective immune cell therapies. Strategies such as genetic modification of immune cells (151), combination therapies with checkpoint inhibitors (134–136), and tumor stromal remodeling hold promise for overcoming these barriers (142–144). Advances in these approaches could significantly improve the therapeutic outcomes for CRC patients undergoing immune cell therapies.

In parallel, rapid advancements in biomedical engineering, synthetic biology, and artificial intelligence (AI), ACT is entering a new era of enhanced precision, efficacy, and safety. Cutting-edge biomaterials and tissue engineering technologies are optimizing immune cells delivery and persistence *in vivo*, improving their functionality within the immunosuppressive tumor microenvironment. Synthetic biology enables fine-tuned regulation of gene-modified immune cells, such as through logic-gated CARs (152) or gene circuit-based controls (153), allowing activation only under specific conditions while mitigating off-target toxicity and immune-related side effects (154, 155). Moreover, artificial intelligence technology (AI) is revolutionizing TCR/CAR therapy by facilitating target selection and leveraging data-driven approaches to refine patient stratification and treatment response prediction (156). Additionally, the combination of CRISPR/Cas9 and AI, particularly in data analysis, optimizing gene editing, studying tumor heterogeneity, CAR-T cell therapy, and clinical trials, can greatly enhance the efficiency of precision medicine and cancer treatment (157).

Looking ahead, the integration of these cutting-edge technologies will drive the development of next-generation ACT with superior therapeutic efficacy and minimized adverse effects. By harnessing precise targeting, programmable regulation, and optimized manufacturing, ACTs hold the potential to overcome existing challenges, achieve durable anti-tumor responses, and provide safer, more effective treatment options for CRC patients.
